# Chloroform Fraction of *Prasiola japonica* Ethanolic Extract Alleviates UPM 1648a-Induced Lung Injury by Suppressing NF-κB Signaling

**DOI:** 10.3390/foods12010088

**Published:** 2022-12-24

**Authors:** Sang Hee Park, Ji Hye Kim, Minkyung Song, Hwa Pyoung Lee, Ji Hye Yoon, Dong Seon Kim, Seok Gu Jang, Dong Sam Kim, Jae Youl Cho

**Affiliations:** 1 Department of Biocosmetics, Sungkyunkwan University, Suwon 16419, Republic of Korea; 2 Department of Integrative Biotechnology, Sungkyunkwan University, Suwon 16419, Republic of Korea; 3 Biomedical Institute for Convergence at SKKU (BICS), Sungkyunkwan University, Suwon 16419, Republic of Korea; 4 Samcheok Prasiola Japonica Research Center, Samcheok City Hall, Samcheok 25914, Republic of Korea

**Keywords:** urban particulate matter, air pollution, lung damage, *Prasiola japonica*, anti-inflammatory, NF-κB

## Abstract

*Prasiola japonica* is an edible alga, and the ethanol extract of *P. japonica* (Pj-EE) possesses various biological activities. Interestingly, in a recent study, we observed the potent anti-inflammatory activity of the chloroform fraction of Pj-EE (Pj-EE-CF). Thus, to extend the application of Pj-EE-CF, we further studied its effects on lung injury. To establish an experimental model of lung injury, we nasally administered urban particulate matter UPM 1648a (50 mg/kg) to mice. In addition, BEAS-2B cells were treated with 300 μg/mL of UPM 1648a for in vitro analysis. Intranasal administration of UPM 1648a increased lung injury score, macrophage infiltration, and upregulation of the inflammatory enzyme inducible nitric oxide synthase (iNOS) in lung tissues. On the other hand, oral administration of Pj-EE-CF (25, 50, and 100 mg/kg) alleviated these pathological features as assessed by lung wet/dry ratio, lung injury score, bronchoalveolar lavage fluid (BALF) protein amount in the lung tissues up to 70%, 95%, and 99%, respectively. In addition, Pj-EE-CF down-regulated the release of inflammatory cytokines, interleukins (ILs), tumor necrosis factor (TNF)-α, and interferon (IFN)-γ elevated by UPM 1648a in the lung tissues and lung BALF up to 95%. According to Western blot and luciferase assay, Pj-EE-CF (100 mg/kg in vivo or 50 and 100 μg/mL in vitro) significantly reduced the nuclear factor-κB (NF-κB) signal activated by UPM 1648a. Finally, UPM 1648a increased cellular reactive oxygen species (ROS) levels in BEAS-2B cells, while Pj-EE-CF reduced them. These results suggest that Pj-EE-CF alleviates UPM 1648a-induced lung damage via anti-inflammatory and antioxidant activities and by suppressing NF-κB signaling. In conclusion, these observations imply that Pj-EE-CF could be a practical component of food supplements to mitigate air pollution-derived lung damage.

## 1. Introduction

Algae, aquatic photosynthetic organisms, contain abundant bioactive compounds such as polyphenols, phycobiliproteins, and vitamins with numerous medicinal effects, including antioxidant, anticancer, and antiviral properties and are of interest in the pharmaceutical industry [1]. Algae (especially chlorophyte and Bryophyta algae) are a valuable source of dietary supplements such as omega-3 polyunsaturated fatty acids (PUFA), β-carotene, astaxanthin, and carotenoids [1]. *Prasiola* is a genus of leafy green algae that inhabit freshwater, terrestrial, and marine environments. A total of 36 species of the genus *Prasiola* have been reported, of which 14 are freshwater species [2]. In Korea, Park et al. found a *Prasiola* species in Samcheok, Gangwon-do, in 1970 [3], and this was later identified as *P. japonica*, distributed in Korea and Japan as traditionally edible algae [4]. Pharmaceutical benefits such as antioxidant, anti-inflammatory, and skin-protective effects have been confirmed in various in vitro and in vivo models [5,6,7].

In a recent study, we compared the general anti-inflammatory effects of solvent fractions of Pj-EE prepared with n-hexane, chloroform, n-butanol, and water [8]. Interestingly, the chloroform fraction (Pj-EE-CF) was most effective in suppressing nitric oxide levels and inflammatory cytokine gene expression in LPS-stimulated macrophages and in reducing edema in carrageenan-treated paws [8]. The predominantly used indicators in the evaluation of the inflammatory activities of compounds or plant-derived extracts are influenced by Pj-EE [9,10,11]. Thus, we further studied the application of Pj-EE-CF in other inflammation-related diseases in this study. Among many diseases, we examined a model of lung disease, which has become a serious problem in Korea due to the explosive accumulation of air pollution, including particulate matter [12,13].

Air pollution is a major health threat worldwide. Numerous published works indicate that exposure to air pollution is associated with increased respiratory and vascular disease and leads to high morbidity and mortality [14,15]. The components of air pollution vary depending on the source but mainly include particulate matter (PM), nitrogen dioxide (NO_2_), sulfur dioxide (SO_2_), and ozone (O_3_) [16]. Recently, the danger of PM has been emphasized [17]. PM is a mixture of inorganic and organic particles and is classified according to particle size as ultrafine (diameter ≤ 0.1 μm, PM0.1), fine (diameter ≤ 2.5 μm, PM2.5), and coarse particles (diameter ≤ 10 μm, PM10). [18]. PM10 is efficiently deposited in the upper respiratory tract by impaction or gravitational sedimentation [19]. PM2.5, also known as fine dust, can penetrate the alveolar area by diffusion and deposition, affecting the respiratory, cardiovascular, and nervous systems [20]. Furthermore, PM2.5 inhaled into the respiratory tract affects lung macrophages and epithelia [21,22,23,24]. In addition, PM2.5 induces excessive oxidative stresses and reactive oxygen species (ROS)-dependent systemic inflammation [25,26]. Moreover, epidemiological works have shown that PM2.5 increases the risk of *Pseudomonas aeruginosa* (*P. aeruginosa*) infection and pneumonia [21,22,24]. Despite these harmful effects, studies on molecular mechanisms and methods to prevent and reduce PM-derived health problems are limited. Urban particulate matter (UPM) 1648a is a commonly used material for in vivo and in vitro experimental studies regarding exposure to air pollution. According to the literature [27,28], UPM 1648a impairs the cardiovascular system and skin barrier function and causes oxidative stress. In addition, UPM 1648a has been reported to exacerbate arthritis and induce hyperinflammatory responses [29,30]. In our study, nasal administration of UPM 1648a also increased the levels of cytokines in the lung tissues and BALF, leading to lung injury. This evidence indicates that the UPM 1648a-induced lung injury model is suitable for testing the anti-inflammatory effect of Pj-EE-CF. Therefore, we evaluated the health benefits of Pj-EE-CF using this model and also evaluated the anti-inflammatory mechanism of Pj-EE-CF against lung inflammation caused by UPM1648a using the BEAS-2B cell line (a human bronchial epithelial cell line).

## 2. Materials and Methods

### 2.1. Materials

BEAS-2B cells (ATCC number CRL-9609) were purchased from American Type Culture Collection (ATCC) (Rockville, MD, USA). UPM 1648a (NIST SRM 1648a) was obtained from the National Institute of Standards and Technology (NIST, USA). According to the certificate of analysis provided by the NIST, UPM 1648a was collected in the St. Louis, MO area over a certain period (1976–1977). Collected materials were combined into a single lot, and extraneous materials were removed through a fine-meshed sieve and then blended with a V-blender. The composition and homogeneity of UPM 1648a are continuously monitored by the NIST for quality assurance. Dimethyl sulfoxide (DMSO), sodium dodecyl sulfate (SDS), and 3-(4,5-dimethylthiazol,2-yl)-2,5-diphenyltetrazolium bromide (MTT) were purchased from Sigma-Aldrich Co. DMEM, penicillin–streptomycin, trypsin, and phosphate-buffered saline, were purchased from HyClone (Logan, UT, USA). Fetal bovine serum (FBS) was obtained from Biotechnics Research, Inc. (Irvine, CA, USA). TRIzol reagent was purchased from MRCgene (Cincinnati, OH, USA). The enzyme-linked immunosorbent assay (ELISA) kits for IL-1β (MLB00C), IL-6 (M6000B), TNF-α (MTA00B), IL-4 (M4000B), IL-12 (M1270), and IFN-γ (MIF00) were obtained from R&D Systems (Minneapolis, MN, USA). Cell lysis buffer and phospho-specific or total-protein antibodies against IκBα, p-50, p65, and β-actin were obtained from Cell Signaling Technology (Beverly, MA, USA).

### 2.2. Pj-EE and Fraction Preparation

**Figure 1:**
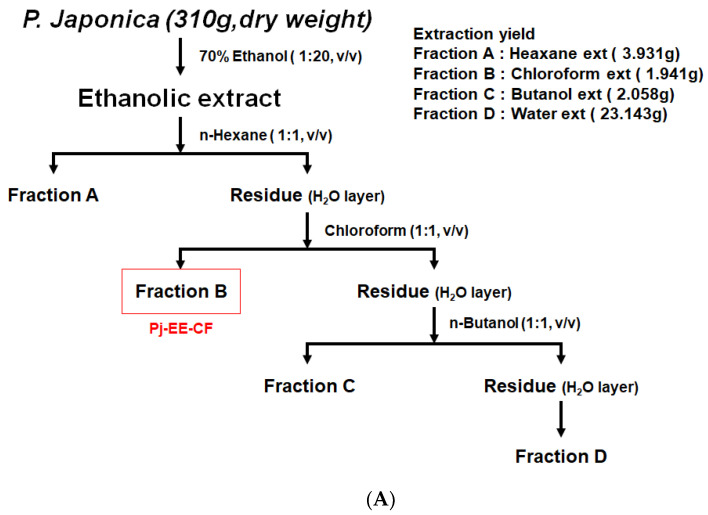

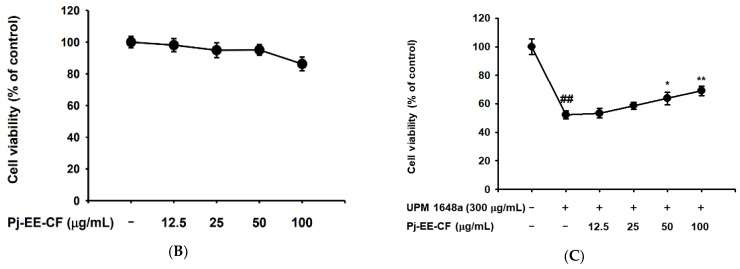
Cytotoxicity and cytoprotective effects of Pj-EE-CF in *BEAS-2B* human bronchial epithelial cells. (**A**) Fractionation diagram of the various solvents of *P. japonica* ethanolic extract (Pj-EE). (**B**,**C**) BEAS-2B cells were treated with UPM1648a (300 μg/mL) and Pj-EE-CF (0–100 μg/mL) or Pj-EE-CF (0–100 μg/mL) alone for 24 h. Cell viability was analyzed analytically by MTT. Data in (**B**,**C**) are presented as mean ± SD of six replicates (*n* = 6). ## *p* < 0.01 compared to normal (non-treatment), * *p* < 0.05 and ** *p* < 0.01 compared to control (UPM 1648a alone).

*P. japonica* was provided by the *Prasiola japonica* Research Center (Samcheok City, Gangwon-do, Republic of Korea). First, the dried sample was cut into 1 mm samples, and 70% ethanol was added at a ratio of 1:20 (*w*/*v*) to extract for 24 h. Then, the supernatant, excluding the precipitate, was filtered using a 110 nm filter paper (No. 2, Advantec, Toyo Co., Tokyo, Japan), and ethanol remaining in the solution was removed through a vacuum concentrator (Eyela New Rotary Vacuum Evaporator, Rikakikai Co., Tokyo, Japan). Finally, the sample was dried by a vacuum freeze dryer (Eyela FD1, Rikakikai Co., Tokyo, Japan) for 72 h [31,32]. The total sample weight was 310 g, the extracted amount was 33.143 g, and the yield was 10.69%. As shown in Figure 1A, the ethanol extract of *P. japonica* was fractionated using n-hexane, chloroform, n-butanol, and water. The yields of these preparations were 1.27% (hexane fraction), 0.63% (chloroform fraction), 0.67% (butanol fraction), and 7.47% (water fraction). The dried samples were stored in a −20 °C freezer.

### 2.3. Cell Culture and Cell Viability Assay

Human bronchial epithelial BEAS-2B cells were cultured in DMEM containing 10% FBS, 100 mg/mL streptomycin, and 100 U/mL penicillin at 37 °C in a 5% CO_2_ humidified incubator. BEAS-2B cells (5 × 10^4^ cells/mL) were seeded in a 96-well plate and treated with Pj-EE-CF (0–100 μg/mL) for 24 h. To test the cytoprotective activity of Pj-EE-CF (0–100 μg/mL), we treated BEAS-2B cells with Pj-EE-CF and UPM 1648a (300 μg/mL) or UPM 1648a (300 μg/mL) alone for 24 h. A conventional MTT assay determined cell viability and cytoprotective activity [33,34].

### 2.4. Animals

ICR mice (8 weeks old, male, 20–21 g) were purchased from Orient Bio (Sungnam, Korea) and bred at SKKU animal holding facility. Breeding facilities are pathogen-free, maintain a constant temperature (21–23 °C) and constant humidity (45–60%), and maintain a 12 h light/dark cycle. The mice were divided into five study groups, the control (vehicle) group, UPM1648a (50 mg/50 μL) group, and three groups representing UPM 1648a exposure (50 mg/50 μL) + PJ-EE-CF (25, 50, and 100 mg/kg), with five mice per group. The control mice were orally administered saline. UPM mice were intranasal administration with 50 μL of PBS containing 50 mg/mL UPM1648a for 3 days. For accurate intranasal administration, all mice were anesthetized just before intranasal administration. Mice in the UPM + PJ-EE-CF groups were given Pj-EE-CF (25–100 mg/kg) orally twice per day for three days, once an hour before UPM1648a treatment and once an hour after UPM1648a treatment. Mice were sacrificed after three days. BALF and lung samples were isolated. BALF was immediately collected, and all lobes of each lung were harvested. The collected BALF was used for ELISA analysis, and the largest left lobe of the lung was used for wet/dry ratio analysis. The middle and lower lobes were used for histopathological data, and the upper lobes were used for Western blotting. All animal experiments were performed in accordance with the guidelines established by the Institutional Animal Care and Use Committee (IACUC) of Sungkyunkwan University (IACUC No.: SKKUIACUC2021-04-12-1).

### 2.5. Lung Wet-to-Dry Weight Ratio and Protein Concentration Ratio Measurement

The left lobes of mouse lung tissue were washed with PBS. After recording the wet weight, the lung tissue was dried using an oven at 60 °C for 72 h, and the weight of the dried lung tissue was measured. Wet-to-dry ratios were calculated to assess the degree of inflammation in the lung tissue [35,36]. The protein concentration ratio was analyzed using the collected BALF. The protein concentration of the collected BALF was analyzed using the Bradford protein quantification method, and the analyzed result was quantified using a protein concentration standard curve. A protein concentration standard curve was determined by dissolving BSA (0–4 mg/mL) in PBS.

### 2.6. Histological Analysis of Lung Tissue

The right lobes of the mouse lung were harvested and fixed in 4% formalin solution for 2 days. The fixed samples were embedded with paraffin, cut to a thickness of 3 μm, and then stained with Hematoxylin & Eosin. Lung injury was assessed by analyzing septal thickening of the alveolar walls, neutrophil infiltration, and membrane structure formation composed of cell debris according to a previously published paper [36,37] and as described in Table 1.

### 2.7. ELISA in BALF and Lung Tissue Lysate

Then, 500 μL BALF was extracted from the trachea of each mouse with 100 μM EDTA in 1 mL PBS. It was prepared at the same concentration for each group (*n* = 5/group) by adjusting with PBS based on Bradford assay. To obtain the lung tissue lysates, lung tissue was lysed by treating cell lysis buffer and homogenized with sonicator [36]. Tissue lysates were centrifuged at 11,000× *g* for 5 min at 4 °C, and supernatants were used for ELISA. Protein concentrations of IL-1β, IL-4, IL-6, IL-12, IFN-γ, and TNF-α in BALF and lung tissues were determined according to the manufacturer’s instructions.

### 2.8. Whole-Cell Lysate Preparation and Western Blotting Analysis

Lung tissue was lysed with cell lysis buffer and sonicated for whole cell lysates. Cell lysates obtained by homogenization were centrifuged at 11,000× *g* for 5 min at 4 °C, and supernatants were used for Western blotting analysis. Protein samples were separated by protein size through SDS-polyacrylamide gel electrophoresis. The gel containing the proteins was transferred to a polyvinylidene fluoride (PVDF) membrane. The first antibody was attached to total and phosphorylated proteins. A secondary antibody recognizing the first antibody was added. It was visualized using an enhanced chemiluminescence reagent.

### 2.9. Luciferase Reporter Gene Activity

Regarding the luciferase reporter assays, baes-2b cells (2 × 10^5^ cells/mL in 12-well plates) were transfected with 1 μg of plasmid-containing β-galactosidase and NF-κB-1-Luciferase reporter gene using lipofectamine 2000 (Thermo Fisher Scientific, Waltham, MA, USA). The cells were incubated with Pj-EE-CF (0–100 μg/mL) and UPM 1648a (300 mg/mL) or UPM 1648a (300 μg/mL) alone for 24 h. The cells were lysed using a cell lysis buffer reacted with luciferin to generate fluorescence, and then fluorescence was measured using a luminescence spectrophotometer. Normalization of the luciferase reporter assay was performed through the activity of β-galactosidase [38].

### 2.10. Cellular ROS Assay

BEAS-2B cells were dispensed in a 12-well plate to be 1.5 × 10^5^ cells/well and cultured using a 5% CO2 incubator for 24 h. Cells were treated with Pj-EE-CF (0–50 μg/mL) and UPM 1648a (300 μg/mL) or UPM 1648a (300 μg/mL) alone. After 24 h, the cultured cells were washed three times with PBS and stained with H2DCF-DA (10 μM). The stained cells were analyzed using a CytoFLEX Flow Cytometer, and fluorescence was analyzed (Beckman Coulter Life Sciences, Indianapolis, IN, USA) [32,39].

### 2.11. Statistical Analysis

**Figure 2:**
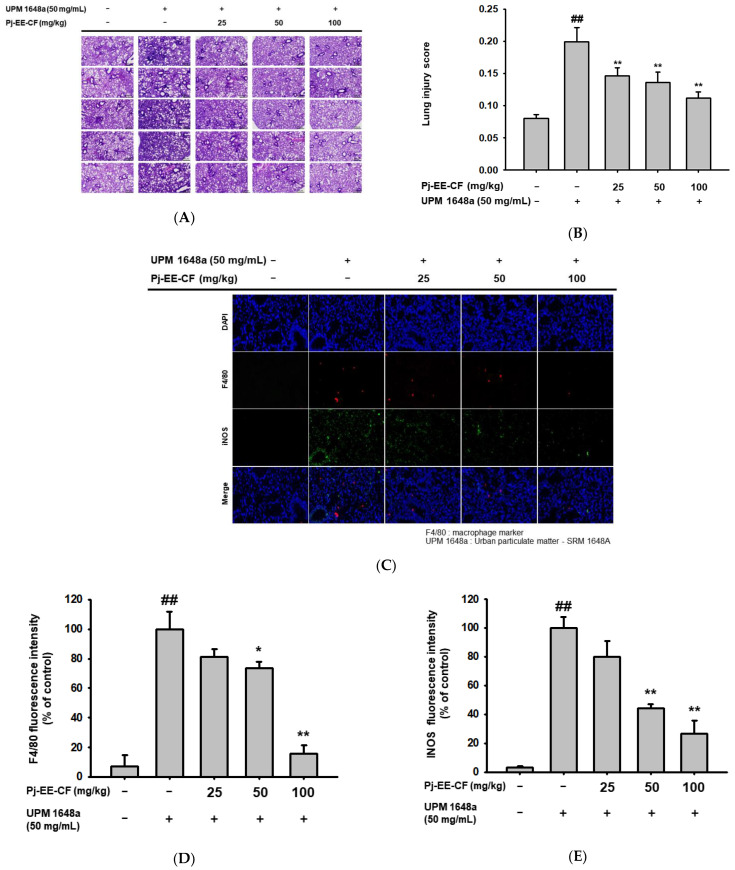

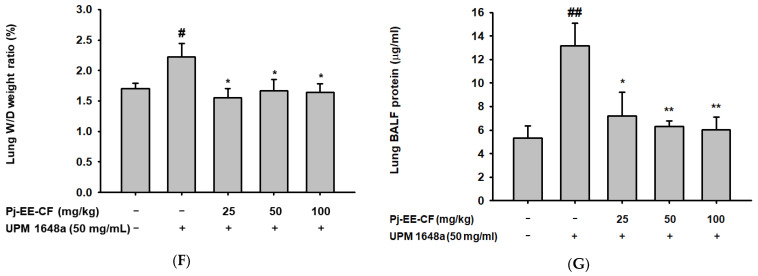
Pulmonary pathological alteration after UPM 1648a instillation and Pj-EE-CF administration in mice. (**A**) Representative image of the pathologic features of lung tissues prepared with five mice per group. Lung tissues from UPM 1648a and Pj-EE-CF treated mice were H&E stained. (**B**) Lung injury score of pulmonary tissue in each group. (**C**) A fluorescence microscopy image of macrophage and iNOS in lung tissue. Immunofluorescence was employed to assess macrophage infiltration (F4/80: red) and iNOS (green), and nuclei were stained with DAPI (blue). (**D**,**E**) Quantification of fluorescence intensity. Fluorescence intensities of F4/80 and iNOS were analyzed using Image J software and fluorescence intensities relative to control were calculated as percentage and expressed as mean ± SD.(**F**) Lung wet/dry (W/D) ratio in each group. The pulmonary water content was investigated by analyzing the lung W/D ratio. (**G**) Protein concentration in BALF prepared from UPM 1648a-exposed mice orally pretreated with Pj-EE-CF (25–100 mg/kg) was determined by the Bradford assay. All assays depicted in (**A**–**G**) were performed with five mice per group. Results (**B**,**D**–**G**) are presented as mean ± SD. # *p* < 0.05 and ## *p* < 0.01 compared to normal (non-treatment), * *p* < 0.05 and ** *p* < 0.01 compared to control (UPM 1648a alone).

**Figure 3:**
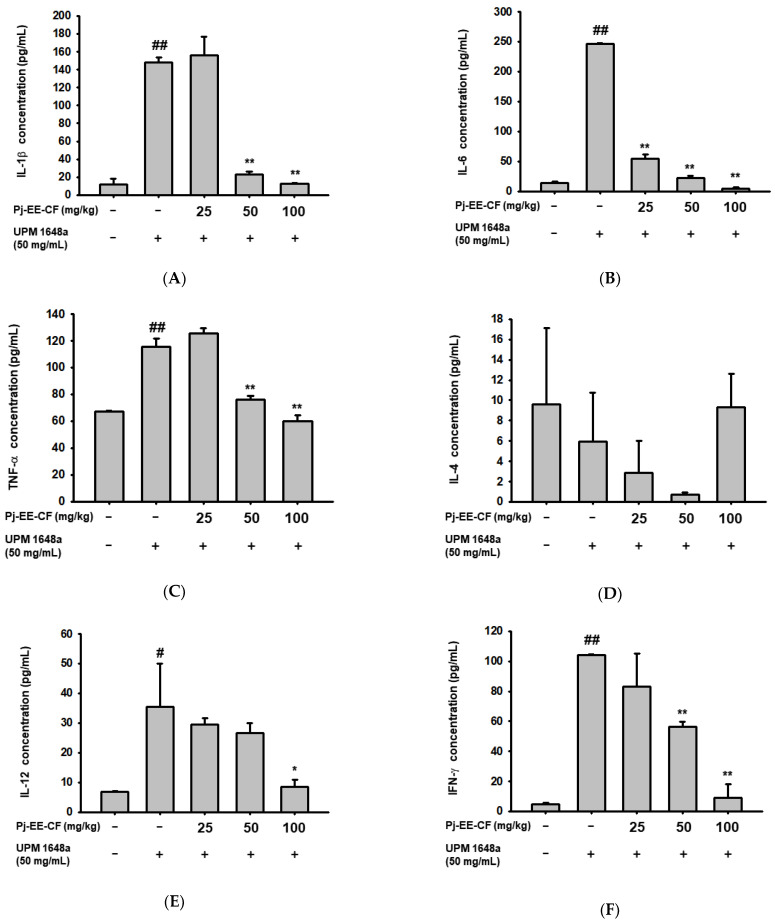
Inflammatory cytokine levels in BALF after UPM 1648a instillation and Pj-EE-CF administration in mice. IL-1β (**A**), IL-6 (**B**), TNF-α (**C**), IL-4 (**D**), IL-12 (**E**), and IFN-γ (**F**) concentrations were determined by ELISA with BALF, using the same amount of protein as adjusted with PBS. All data are presented as mean ± SD of five biological replicates (n = 5 mice/group). # *p* < 0.05 and ## *p* < 0.01 compared to normal (non-treatment), * *p* < 0.05 and ** *p* < 0.01 compared to control (UPM 1648a alone).

**Figure 4:**
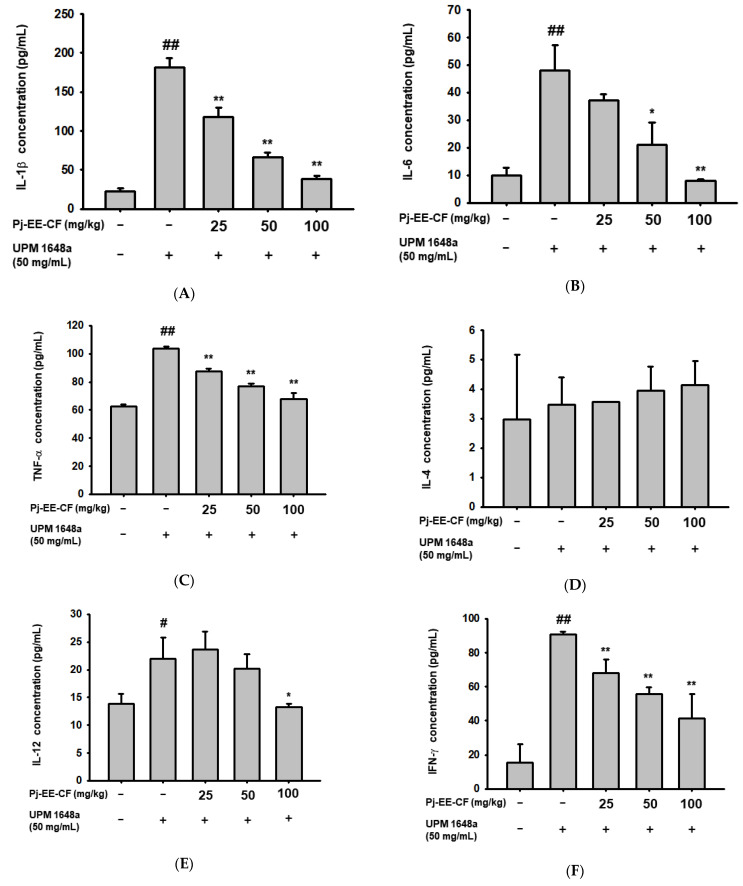
Inflammatory cytokine levels in mouse lung tissue homogenates after UPM 1648a instillation and Pj-EE-CF administration. IL-1β (**A**), IL-6 (**B**), TNF-α (**C**), IL-4 (**D**), IL-12 (**E**), and IFN-γ (**F**) concentrations were determined by ELISA with lung lysates of 5 mice. All data are presented as mean ± SD (standard deviation) of the five biological replicates (n = 5 mice/group). # *p* < 0.05 and ## *p* < 0.01 compared to normal (non-treatment), * *p* < 0.05 and ** *p* < 0.01 compared to control (UPM 1648a alone).

**Figure 5:**
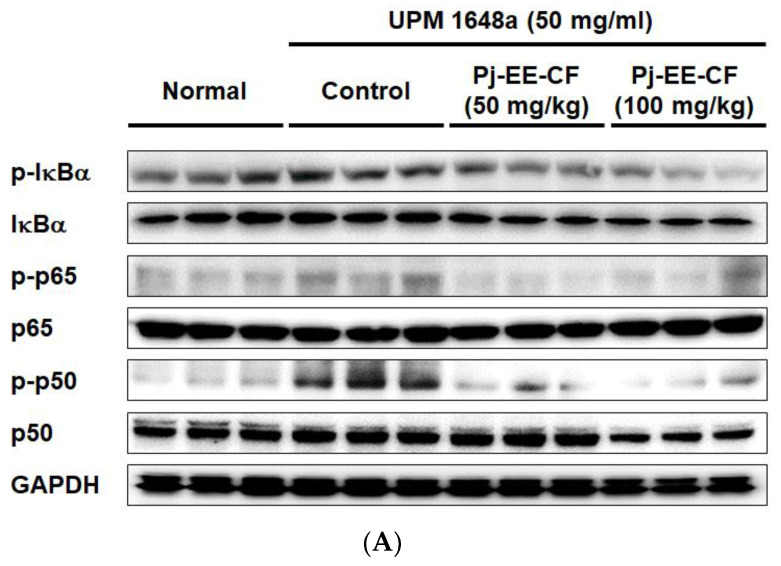

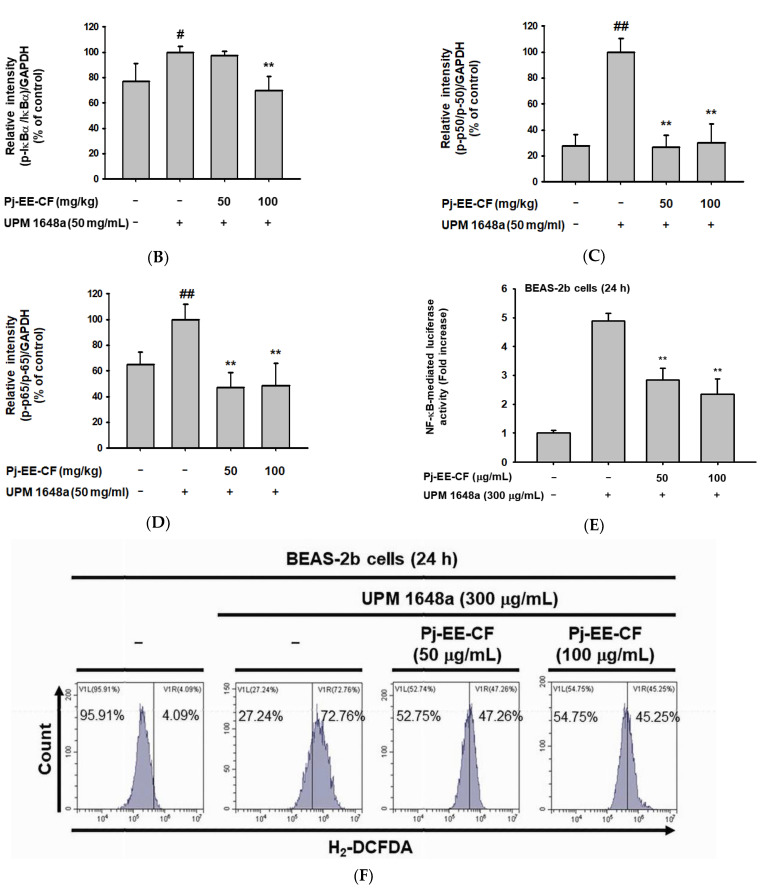
Inhibition of NF-κB signal by Pj-EE-CF. (**A**) Immunoblots of NF-κB signal molecules (Iκba, p65, p50) in UPM 1648a- and Pj-EE-CF-treated mouse lung tissues. Immunoblot images show all three biological replicates. NF-κB pathway-related molecules were detected using antibodies for total and phospho-forms of IκBα, p50, and p65. (**B**–**D**) Band intensity of the immunoblots was measured and quantitated through Image J software, and the relative intensity of the band is expressed as mean ± SD of the three biological replicates (n = 3 mice/group). (**E**) NF-κB luciferase assay in BEAS-2B cells treated with UPM 1648a (300 μg/mL) and Pj-EE-CF (0–100 μg/mL) or UPM 1648a (300 μg/mL) alone. Data in (**E**) are presented as mean ± SD of the three samples. (**F**) BEAS-2B cells were treated with UPM 1648a (300 μg/mL) and Pj-EE-CF (0–100 μg/mL) or UPM 1648a (300 μg/mL) alone and labeled with DCFDA. Fluorescence of DCFDA was detected by flow cytometry. # *p* < 0.05 and ## *p* < 0.01 compared to normal (non-treatment), ** *p* < 0.01 compared to control (UPM 1648a alone).

All the results of our study were calculated as mean ± standard deviation (SD) of an experiment performed with six (Figure 1B,C, and Figure 5E), five (Figure 2B,D–G, Figure 3, and Figure 4) or three (Figure 5B–D) replicates per group. Our results were analyzed by ANOVA, Scheffe’s post-hoc test, and Mann–Whitney U test to analyze statistical significance. Results with values less than 0.05 in the analyzed *p*-values were considered statistically significant in all analyses. (# *p* < 0.05, ## *p* < 0.01, * *p* < 0.05, ** *p* < 0.01). All statistical analyses were conducted using the Statistical Package for the Social Sciences program (IBM Corp., Armonk, NY, USA).

## 3. Results

### 3.1. Pj-EE-CF Protects UPM 1648a-Exposed Human Bronchial Epithelium Cells

To evaluate the cytotoxicity of Pj-EE-CF, we treated BEAS-2B cells with Pj-EE-CF (0–100 μg/mL) and performed the MTT assay. Pj-EE-CF did not affect the cell viability of BEAS-2B cells up to concentrations of 100 μg/mL (Figure 1B). The cytotoxicity of UMP1648a (150 μg/mL) has been verified in nasal epithelial cells [40]. Consistently, *BEAS-2B* cell viability was decreased by 50% in the UPM 1648a-treated group (Figure 1C). Interestingly, Pj-EE-CF reversed the *BEAS-2B* cell viability decreased by UPM 1648a exposure, suggesting that Pj-EE-CF can protect bronchial epithelial cells from UPM 1648a-induced cell damage.

### 3.2. Pj-EE-CF Alleviates Pathological Changes of Lung Tissues in UPM 1648a-Stimulated Mice

To analyze the effect of Pj-EE-CF, we stained lung tissues from UPM 1648a-treated mice with H&E. The control group showed a typical pattern of histology, while the UPM 1648a group exhibited histological changes. However, oral administration of Pj-EE-CF (25, 50, and 100 mg/kg) alleviated the histopathological changes (Figure 2A). In parallel, UPM 1648a increased histological injury scores, and Pj-EE-CF decreased them (Figure 2B). In addition, the signal intensity of F4/80, a macrophage marker in lung tissues, was significantly increased by UPM 1648a but decreased by Pj-EE-CF (50 and 100 mg/kg) in a concentration-dependent manner under fluorescence microscopy (Figure 2C,D). Consistently, Pj-EE-CF suppressed UPM 1648a-induced iNOS, an inflammatory enzyme mainly expressed by macrophages (Figure 2C,E). Changes in pulmonary vascular permeability were evaluated by analyzing the W/D ratio of the lung. UPM 1648a increased the lung W/D ratio, and Pj-EE-CF reduced the lung W/D ratio to the control level (Figure 2F). In patients with lung disease, particularly asthma, BALF contains more blood proteins than in healthy people due to plasma extravasation [41]. Likewise, UPM 1648a increased BALF protein content, but Pj-EE-CF administration suppressed the BALF protein concentration (Figure 2G).

### 3.3. Pj-EE-CF Suppresses UPM 1648a-Induced Cytokine Levels in BALF

Next, we analyzed the regulation of Pj-EE-CF on inflammatory cytokines in BALF. The key inflammatory cytokines IL-1β, IL-6, and TNF-α, were significantly increased in BALF by UPM 1648a (Figure 3A–C). Pj-EE-CF (50, 100 mg/kg) reduced UPM 1648a-induced production of IL-1β and TNF-α to the control levels (Figure 3A,C). Pj-EE-CF suppressed the level of IL-6 at all treated concentrations (Figure 3B). Pj-EE-CF and UPM 1648a did not alter the production of IL-4, which exerts dual properties (immunostimulatory and immunosuppressive effects) in lung injury and fibrosis (Figure 3D). In addition, Pj-EE-CF affected IL-12 and IFN-γ, which are important inflammatory cytokines in bacterial pneumonia. UPM 1648a increased IL-12 and IFN-γ, and Pj-EE-CF (0–100 mg/kg) dose-dependently decreased the concentrations of the elevated cytokines (Figure 3E,F).

### 3.4. Pj-EE-CF Decreases UPM 1648a-Induced Cytokine Production in Lung Tissues

As in BALF, treatment with UPM 1648a significantly increased inflammatory cytokines (IL-1β, IL-6, TNF-α, 1L-12, and IFN-γ) in lung tissue. (Figure 4A–C,E,F). Consistent with the results in Figure 3D, IL-4 was not changed by UPM 1648a treatment (Figure 4D). Meanwhile, Pj-EE-CF (0–100 mg/kg) significantly suppressed amounts of IL-1β, IL-6, TNF-α, and IFN-γ in a concentration-dependent manner (Figure 4A–C,F) but did not affect IL-14 level (Figure 4D). In IL-12, only 100 mg/kg of Pj-EE-CF decreased UPM 1648a-induced IL-12 (Figure 4E).

### 3.5. Pj-EE-CF Suppresses NF-κB and Exerts Antioxidant Activity

Since NF-κB has been reported as the primary transcriptional regulator of pro-inflammatory cytokines [42], we further analyzed the effect of Pj-EE-CF on NF-κB signal molecules (Ikba, p65, p50). In resting cells, IκBα blocks NF-κB by binding to it and allowing it to remain in the cytoplasm [43]. Upon external stimulation, IKK phosphorylates IκBα, and the phosphorylated IκBα is degraded. Sequentially, free NF-κB subunits p65 and p50 are phosphorylated, translocated into the nucleus, and act as transcriptional factors. Interestingly, UPM 1648a significantly increased p-IκBα level in lung tissues (Figure 5A,B). On the other hand, Pj-EE-CF (100 mg/kg) inhibited the phosphorylation of IκBα (Figure 5A,B). In addition, the expression levels of p-p50 and p-p65 were upregulated by treatment with UPM 1648a but downregulated by treatment with Pj-EE-CF (50 and 100 mg/kg) (Figure 5A,C,D). We additionally performed a luciferase assay to confirm our hypothesis that Pj-EE-CF affects NF-κB activity. Consistent with the Western blotting results, UPM 1648a increased NF-κB-mediated luciferase activity in *BEAS-2B* cells, whereas Pj-EE-CF (50 and 100 μg/mL) significantly decreased it (Figure 5E). NF-κB has been reported to increase the expression of pro-oxidant genes such as NADPH oxidase NOX2, iNOS, LOX-12, and LOX-5. [44]. Furthermore, LC-MS performed in our previous work showed that Pj-EE-CF contains abundant flavonoids with antioxidant activities [33]. Thus, we assessed the antioxidant activity of Pj-EE-CF. Cellular ROS levels were detected by flow cytometry in combination with dichlorodihydrofluorescein diacetate (DCFDA), a cell-permeable fluorogenic dye for ROS. As shown in Figure 5F, UPM 1648a increased cellular ROS level, but Pj-EE-CF reduced it, suggesting that decreasing ROS levels may be another possible mechanism involved in the effects of Pj-EE-CF.

## 4. Discussion

Prolonged inhalation of UPM causes respiratory diseases, including lung injury, but studies on the precise molecular mechanisms for this and on effective drugs or food supplements are lacking [45,46]. UPM, one of the known air pollutants, is emitted into the atmosphere due to fuel combustion and vehicle exhaust and is also formed in natural forms such as volcanic ash and fire [47]. According to the World Health Organization (WHO) Air Quality Guidelines (AQG), continuous exposure to air pollution increases the incidence of chronic respiratory diseases, strokes, and cardiovascular diseases, especially fine dust (PM10, diameter ≤ 2.5 μm) passes through capillaries to promote inflammatory responses in respiratory system [48]. Furthermore, the WHO’s International Agency for Research on Cancer (IARC) has announced that fine dust is the leading cause of lung cancer. Since UPM varies by season and region, in this study, UPM 1648a, administered by intratracheal instillation, was used to achieve repeatability and high stability [49,50]. UPM 1648a contains endotoxin, metal/nonmetal elements, polycyclic aromatic hydrocarbons (PAHs), and polychlorinated biphenyl homologs [51]. The average diameter of UPM 1648a is approximately 5.86 μm [49], and the size in PBS ranges from 236.43 nm to 1.98 μm [51].

Uncontrolled inflammation is a pivotal pathophysiologic characteristic of acute lung injury [52,53]. Exposure to external stimuli such as PM and LPS induces the secretion of inflammatory cytokines (IL-1β, IL-6) in BALF, leading to inflammatory responses [54,55]. TNF-α is a representative cytokine whose expression rapidly increases in acute inflammation and affects pulmonary diseases (asthma, acute lung injury, acute respiratory distress syndrome) [56,57,58]. IFN-γ elicits Th1-mediated inflammatory responses during acute lung injury, where IL-12 acts as a key upstream regulator of IFN-γ signaling. [59]. Interestingly, UPM 1648a significantly upregulated the cytokines, in particular, IL-1β, IL-6, and IFN-γ in BALF and lung tissue, while Pj-EE-CF dose-dependently suppressed the increased cytokines. TNF-α and IL-12 also showed an increasing pattern upon UPM 1648a exposure and were decreased by Pj-EE-CF.

NF-κB plays a pivotal role in a variety of conditions that promote acute lung injury [60]. For example, leukotriene B4 promotes NF-κB signaling-induced acute lung injury in a single lung ventilation model [61]. UPM 1648a also induces acute lung injury mediating the NF-κB [62]. In addition, the intensity and duration of NF-κB are based on the severity of lung injury in endotoxin-exposed mice [63]. In our previous study, UPM 1648a affected keratinocytes by regulating p38 and NF-κB pathways [64,65]. Other studies have shown that endotoxin present in UPM increases TLR4-mediated inflammatory responses in murine alveolar macrophages [66,67]. Here, NF-κB is one of the significant downstream regulators of TLR4 signaling. It has also been reported that several substances exhibit efficacy in alleviating UPM-induced lung injury through the inhibition of NF-κB and TLR4 [66,68]. Likewise, Pj-EE-CF suppressed UPM 1648a-induced phosphorylation of NF-κB signal molecules (IκBα, p50, p65), alleviating lung inflammation and injury [65,69]. Notably, the inhibitory activity of Pj-EE-CF is so potent that it reduces the p-p50 level increased by UPM 1648a to the basal level. As a result, inhibition of NF-κB led to the suppression of inflammatory cytokines, such as IL-1, -6, -12, TNF-α, and IFN-γ. On the other hand, Pj-EE-CF did not affect the expression of IL-4 expression regulated by the nuclear factor of activated T cells (NF-AT) or c-maf. Considering these results, Pj-EE-CF seems to selectively inhibit NF-κB but not NF-AT.

We previously observed that Pj-EE-CF contains approximately 23 active components and flavonoids, including maltol, bavachinin, kushenol N and X, nobiletin, and phellochinin [8]. Maltol, bavachinin, and nobiletin have various health benefits, including anti-inflammatory, antioxidant, and anti-tumorigenesis effects, and have shown inhibitory activity of NF-κB in inflammation models, such as arthritis and endotoxin shock [70,71,72]. Therefore, the anti-lung injury and NF-κB inhibitory efficacy of Pj-EE-CF might be derived from the synergistic combination of these flavonoids.

Our results explained the pharmacological efficacy of freshwater laver, edible freshwater green algae. However, it is unclear which components in the chloroform fraction would exhibit pharmacological effects. Therefore, based on the previous studies, we will identify which active compound would inhibit NF-κB activity by activity-guided fractionation with chloroform fraction using a luciferase assay system. With this information, we will also compare the level of active compound(s) in other fractions as well as crude ethanol extract using mass spectrometry and HPLC analysis. In addition, further research regarding understanding the mechanisms of therapeutic action and molecular target(s) of the fraction will be followed. Finally, although these green algae have been traditionally used, whether the crude extract or fraction of the green algae have side effects in long-term treatment will be carefully examined.

## 5. Conclusions

In conclusion, Pj-EE-CF mitigated the pathologic features of lung damage, such as lung architecture destruction and lung edema in UPM 1648a-treated mice. In addition, Pj-EE-CF inhibited inflammatory responses via negative regulation of inflammatory cytokine release and macrophage infiltration. Moreover, Pj-EE-CF markedly blocked NF-κB activation induced by UPM 1648a in lung tissues and BALF. Consequently, our results suggest that the Pj-EE-CF fraction can be a pharmaceutical and food supplement to alleviate UPM 1648a-derived pulmonary damage. Since *P. japonica* is edible algae, the effective consumption amount of raw material was 200 to 900 g to reach its effective dose, according to calculations considering the Pj-EE-CF yield. Therefore, additional studies to improve the extraction yield of active ingredients contained in Pj-EE-CF should be continued to develop functional food preparation with the algae.

## Figures and Tables

**Table 1:** Lung injury scoring index [37].

Measurement Criteria	Score		
	0	1	2
A. Neutrophil infiltration into the interstitial space	Not found	1 to 5	More than 5
B. Neutrophil infiltration into the alveolar space	Not found	1 to 5	More than 5
C. Number of hyaline membranes	Not found	3	More than 3
D. Septal thickening of the alveolar wall	More than 2×	2 to 4×	More than 4×
Score = [(20 × A) + (14 × B) + (7 × C) + (2 × D)]/(field number × 100)			

## Data Availability

The data used to support the findings of this study are available from the corresponding author upon request.
